# Socioeconomic Factors and Vulnerability to Outbreaks of Leptospirosis in Nicaragua

**DOI:** 10.3390/ijerph110808301

**Published:** 2014-08-15

**Authors:** Jorge Bacallao, Maria Cristina Schneider, Patricia Najera, Sylvain Aldighieri, Aida Soto, Wilmer Marquiño, Carlos Sáenz, Eduardo Jiménez, Gilberto Moreno, Octavio Chávez, Deise I. Galan, Marcos A. Espinal

**Affiliations:** 1 University of Medical Sciences of Habana, Havana Atherosclerosis Research and Reference Center, Policlínico 19 de Abril, Tulipán y Panorama, Nuevo Velado, Plaza de la Revolución, La Habana 10600, Cuba; 2 Pan American Health Organization, Department of Communicable Diseases and Health Analysis, 525 23rd. St. NW, Washington, DC 20037, USA; E-Mails: najerapa@paho.org (P.N.); aldighsy@paho.org (S.A.); deisegalan@gmail.com (D.I.G.); espinalm@paho.org (M.A.E.); 3 Pan American Health Organization Nicaragua, P.O. Box 1309, Managua, Nicaragua; E-Mails: sotoa@nic.ops-oms.org (A.S.); marquinw@nic.ops-oms.org (W.M.); 4 Ministry of Health of Nicaragua, Costado Oeste Colonia Primero de Mayo, P.O. Box 107, Managua, Postal Sector 15AB, Nicaragua; E-Mails: carlossaen@gmail.com (C.S.); zoonosis@minsa.gob.ni (E.J.); ggmavellan@yahoo.com (G.M.); ve1s67-chi@minsa.gob.ni (O.C.)

**Keywords:** leptospirosis, vulnerability index, socioeconomic factors, risk, outbreaks, Nicaragua

## Abstract

Leptospirosis is an epidemic-prone zoonotic disease that occurs worldwide, with more than 500,000 human cases reported annually. It is influenced by environmental and socioeconomic factors that affect the occurrence of outbreaks and the incidence of the disease. Critical areas and potential drivers for leptospirosis outbreaks have been identified in Nicaragua, where several conditions converge and create an appropriate scenario for the development of leptospirosis. The objectives of this study were to explore possible socioeconomic variables related to leptospirosis critical areas and to construct and validate a vulnerability index based on municipal socioeconomic indicators. Municipalities with lower socioeconomic status (greater unsatisfied basic needs for quality of the household and for sanitary services, and higher extreme poverty and illiteracy rates) were identified with the highest leptospirosis rates. The municipalities with highest local vulnerability index should be the priority for intervention. A distinction between risk given by environmental factors and vulnerability to risk given by socioeconomic conditions was shown as important, which also applies to the “causes of outbreaks” and “causes of cases”.

## 1. Introduction

Leptospirosis is among the most common bacterial infections transmitted from animals to humans and has a significant health impact in many parts of the world, affecting primarily vulnerable populations. According to the Leptospirosis Burden Epidemiology Reference Group (LERG), it is considered a neglected disease and further investigation is needed of its epidemiological distribution, total burden of disease and contributing factors [1]. Leptospirosis is geographically disseminated worldwide and it is estimated that over 500,000 human cases occur annually, with a case fatality rate ranging from <5% up to 30% [2]. Various animals, among which includes cattle, pigs, dogs, rats and other peridomestic rodents, have been identified as maintenance hosts of *Leptospira*. Humans can become infected through direct contact with urine of infected animals or by indirect exposure to contaminated objects or environments [2].

Leptospirosis is one of the most illustrative examples, among other infectious diseases, of the effects of the interaction between humans and their physical and social environment, and particularly in the interface with animals and the environment. These interactions may be altered by the effects of climate change, which may affect the frequency of outbreaks and intensity of infection [3]. The “One health” framework, an integrated approach between public health, animal health and the environment, can be used to improve the understanding of this disease and to develop control strategies. 

Environmental drivers that influence the epidemiology of leptospirosis include high temperatures, rainfall and floods, which cause exposure to animal hosts and to precarious situations of lack of clean water and basic sanitation [1,4,5,6]. Urbanization and climate change can cause an increase in the frequency and intensity of these factors, consequently increasing the incidence of the disease and the occurrence of outbreaks [3]. Other studies have also found an association between soil types, particularly in relation to its ability to retain moisture, and the levels of proliferation of leptospira [4,7]. Alkaline and neutral soil types, especially of volcano origin, may facilitate longer survival of the bacteria [4,8]. 

Socioeconomic factors may also influence the risk of acquiring the disease. Proximity to pig farms, low income, outdoor occupations and inadequate health education have been shown to be contributing factors for increased risk of leptospirosis infection [9]. In addition, studies conducted at urban slums concluded that close contact with garbage and sewage, as well as low socioeconomic status are risk factors for acquiring leptospirosis [3,10,11,12,13]. Further, a positive correlation between *Leptospira* infection and low educational level has been found [14]. 

According to the World Health Organization “the bulk of the global burden of disease and the major causes of health inequities, arise from the conditions in which people are born, grow, live, work, and age”, referred to as social determinants of health [15,16]. Conditions such as inadequate access to safe drinking water and sanitation services, coupled with poor personal hygiene practices, low levels of literacy, gender inequality, inadequate nutrition and lack of access to health services, contribute to increased vulnerability to infection and work against prevention efforts. The Rio Political Declaration on the Social Determinants of Health that was adopted by WHO Member States in 2011, as well as the Finland Statement on Health in All Policies that was agreed upon in June 2013 reinforce the importance of engaging all sectors of government, all segments of society, and all members of the international community in addressing these social determinants [17,18]. Understanding the relationship between leptospirosis and socioeconomic indicators may demonstrate the importance of identifying and addressing the social determinants in leptospirosis prevention programs. 

These evidences demonstrate the importance of the combination of two factors that may affect the incidence and lethality of the disease. First, factors associated with the physical environment (rainfall, floods, soil types, presence and distribution of animals), which are generally difficult to modify or not modifiable at all. Second, factors related to socioeconomic conditions, which form the fundamental scenario through which the environmental factors are exacerbated. Possibly, environmental factors establish the conditions for the occurrence of outbreaks and socioeconomic conditions influence the incidence of cases. 

Education stands as a factor of particular importance among those indicators of socioeconomic conditions. It has been well established as a predictor of risk for many diseases but also as one of the factors with greater impact on public health indicators, when it is the focus of public health interventions or programs [19].

In Nicaragua several conditions converge and create an appropriate scenario for the development of leptospirosis risk factors, from its environmental characteristics especially suitable for the occurrence of outbreaks, to its large socioeconomic gradient and indicators that demonstrate social vulnerability [4,20]. The country has also experienced natural disasters that are normally identified as antecedents for outbreaks [21,22]. Several leptospirosis outbreaks have been documented in Nicaragua, with one of the most significant occurring after hurricane “Mitch” in 1998, with 2259 clinical cases documented in the literature [21,22,23]. On the other hand, Nicaragua has a timely surveillance system with community participation using an inter-institutional and intersectoral approach, and has accumulated experience in the development of local capacities for disease control [24]. 

Until 2000–2002, the diagnosis of leptospirosis was based on clinical management and laboratory confirmation through the micro-agglutination test (MAT), which is considered the gold standard by WHO, a technique only available at Nicaragua’s National Center for Diagnosis and Reference [2,4]. As of 2003, active surveillance of cases with fever was initiated, using a standardized ELISA test available in laboratories of the national network present in all departments. In 2008 and 2009, active surveillance was put in place to detect cases using the techniques mentioned above. Since 2010, besides using ELISA as a screening method, a rapid test is performed at local level, which enhances early detection and the management of outbreaks at primary care level. Samples are sent to the Central Laboratory to confirm the diagnosis using MAT. Nicaragua’s national health policy is based on the Family and Community Health Care Model, a program free of charge provided by the government to all citizens, including the most poor and those in remote areas. This system is instituted throughout the country and includes coverage of primary care and surveillance actions, which allows outbreaks and cases to be timely reported, in addition to recording data that can be used for evidence based studies for decision making. 

In a previous study conducted in Nicaragua, an analysis was carried out to stratify the risk and identify “critical areas” for leptospirosis outbreaks, as well as perform an exploratory analysis of potential “drivers” [4]. In this study, incidence rates were mapped for the entire country and also some variables considered as potential “drivers”. An association was found between outbreaks and environmental variables that have a greater presence in the Pacific Region of Nicaragua. In addition, most of the critical zones (higher risk areas) identified and half of the human cases reported were located in three departments (Leon, Chinandega and Managua) on the Pacific Coast. This information served as basis for the design of a second study. 

The objectives of this study are to explore possible socioeconomic variables related to leptospirosis critical areas and to construct and validate a vulnerability index based on municipal socioeconomic indicators. This index could be used as a criterion for targeting resources and actions to prevent and respond to leptospirosis outbreaks in Nicaragua and other countries critical areas of Central America and the Caribbean. 

## 2. Material and Methods 

### 2.1. Study Design and Data

This ecological study was conducted by municipality (second sub-national level) and included all the 32 municipalities of the three departments (first sub-national level) with the highest number of cases of leptospirosis in Nicaragua: Chinandega, Leon, and Managua. In addition, these departments together reported 1001 human cases that represented 50.6% of all cases of leptospirosis reported in Nicaragua between 2004 and 2010, time period of the previous study that identified the critical areas using the same database with country official information [4]. Chinandega and Leon have been identified by local experts and in the previous study as risk areas for having the highest incidence rates among all municipalities. Managua, the capital city, also contributed to the statistics with a substantial number of cases. These three departments, with a population of 2,056,745 inhabitants (39.09% of the total country population), are located in the Pacific Coast where the majority of the municipalities are considered critical zones for outbreaks of leptospirosis and where there is a high frequency of some of the environmental driving factors that were identified in a previous study in Nicaragua [4]. 

Human cases of leptospirosis detected by the country’s Ministry of Heath surveillance system were recorded and the study time period was defined (2004–2010). Individual cases of leptospirosis with laboratory confirmation are included in the national surveillance system, for the purpose of this study the cases were aggregated by municipalities and incidence rates were estimated. The selected variables, sources scale and range of information used in this study are summarized in Table 1. The socioeconomic variables were obtained from secondary data from Nicaragua’s census and United Nation’s Economic Commission for Latin America and the Caribbean (ECLAC) (in Spanish, Comisión Económica para América Latina y el Caribe, CEPAL) [20,25]. No secondary data was available to study individual cases and their socioeconomic status. The ranges of most variables are large because the study includes the national capital, as well as small municipalities with mostly rural population.

**Table 1:** Variables and sources of information used in the study.

Variables	Sources	Scale	Range
Cases of Leptospirosis 2004–2010	Ministry of Health. Nicaragua [26]	Number of cases	0–176
Population of Municipality 2005	Nicaragua Census & annual projections [20]	Number of people	4719–937,489
UBN Quality of the Household	CELADE, CEPAL [25]	Percentage	20.07–83.38
UBN Access to Sanitary Services	CELADE, CEPAL [25]	Percentage	5.27–55.04
UBN Crowding	CELADE, CEPAL [25]	Percentage	8.70–52.60
UBN Access to Education	CELADE, CEPAL [25]	Percentage	3.92–16.45
People in Municipality Living in Extreme Poverty	Nicaragua Census [20]	Percentage	15.7–53.5
Population of 6-yr-olds and Over, Condition of Illiteracy in Municipality	Nicaragua Census [20]	Percentage	7.91–26.25
Household with Piped Water	CELADE, CEPAL [25]	Percentage	0.66–80.01
Household with Solid Waste Disposal	CELADE, CEPAL [25]	Percentage	0.31–83.69

Note: UBN: Unsatisfied Basic Needs.

The socioeconomic variables analyzed in this study were: unsatisfied basic needs (UBN), percentage of population living in extreme poverty, piped water in the household, solid waste disposal in the household and illiteracy rates in the population over 6 years old. 

“Unsatisfied basic needs” is a direct indicator of poverty used by Latin American countries that was developed in the late 1970’s by ECLAC [27]. UBN measures the goods and services associated with the wellbeing that a household possesses. It verifies whether households have satisfied a number of previously established needs or deficiencies, considering poor those who have failed [25]. The UBN used in this study are divided into three general categories: housing conditions, access to sanitary services and access to education, which are described in detail below [27]:

- Housing conditions were measured using two indicators: (1) UBN for quality of the household verifies the construction materials used in the floor, walls and roof; (2) UBN for crowding measures the size of the house in relation to the number of people living there.
- Access to sanitary services determines the availability of potable water and the system for elimination of human excreta.
- Access to education measures enrollment of school-age children in school.

### 2.2. Analysis

Incidence rates were calculated for 10 thousand habitants and logarithmically transformed using the expression below to reduce the asymmetry of the distribution:

*Z*_t_ = ln (1 + *rates*)

A constant was added to avoid the indefinability of the logarithmic function at zero rates. From the distribution of log-transformed rates three groups were defined as follows:

1. Group I (low rates): 0.0 ≤ Z_t_ ≤ 0.50
2. Group II (medium rates): 0.51 ≤ Z_t_ ≤ 1.00
3. Group III (high rates): Z_t_ > 1.00

Descriptive statistics for the three clusters of municipalities were calculated for the socioeconomic variables. The clusters are composed of municipalities and the municipalities are characterized by socioeconomic indicators which are used to identify the clusters. A two-step cluster analysis was conducted using the socioeconomic indicators. The classification generated by the clustering algorithm was tested for concurrent validity by means of the crude leptospirosis rates [28]. Concurrent validity is defined as the association between two different criteria which are concurrently measured on a set of objects (to distinguish it from predictive validity) [29]. The relative importance of the variables (RIV) for the definition of clusters was then computed as:

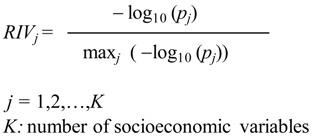

where *p_j_* is the *p* value associated with the Fisher F statistic corresponding to a one-way analysis of variance (ANOVA) between clusters, in which variable *j* is the dependent variable (one *p* value for every one-way ANOVA performed was obtained to assess how well a particular variable discriminates between clusters). The ANOVA’s were performed between clusters of municipalities by using one socioeconomic indicator at a time. Each ANOVA yields a *p* value which constitutes a proxy measure of how well that particular socioeconomic indicator separates the clusters. Log (1) is 0 and log of numbers <1 are negative, which explains the minus sign in the expression. Thus, the numbers in the numerator and denominator of the above expression are positive and their quotient is a number between 0 and 1, extremes not included. 

RIV_j_ values were then used as weights to construct a local vulnerability index (LVI) to be applied in risk areas and in outbreak situations, with the purpose of identifying vulnerable municipalities or geo-demographic areas [30]. The LVI is a latent variable defined as the linear combination of the socioeconomic indicators, taking into account the polarity of the variables (positive or negative indicator) in the allocation of the signs. Thus, the LVI could be obtained as a combination of the variables in which the relative importance or coefficient is used as the weighed factor. In addition, the LVI is a nonspecific index that after proper validation could also be used for similar infectious diseases:

$$LVI=\sum_{j=1}^{K}RIV_{j}\times SEV_{j}$$

SEV_j_ stands for the value of the socioeconomic variable *j* (*j* = 1,2,…,K) and RIV_j_ is the relative importance of variable j, as described previously. A LVI was calculated for each municipality. Concurrent validity of the index was assessed in relation to the previously described municipal classification. As part of this analysis of the concurrent validity of LVI a classification tree was adjusted that helped define cutoff points for qualitative diagnosis of vulnerability in terms of adjusted risk. Cut-offs that maximize the association between the classification of the municipalities based on the vulnerability index and the classification based on incidence rates was chosen. 

## 3. Results and Discussion

Table 2 shows the linear correlation coefficients between log-transformed rates and the socioeconomic variables. Highly significant associations were obtained for all indicators.

**Table 2:** Pearson correlation coefficients (and *p* values) between log-transformed rates and socioeconomic variables at the municipality level (*n* = 32 municipalities).

Socioeconomic Variable	Pearson Correlation	*p* Value
UBN Quality Household	0.525	0.002
UBN Sanitary Services	0.686	0.000
UBN Crowding	0.609	0.000
UBN Education	0.606	0.000
Extreme Poverty	0.610	0.000
Illiteracy	0.652	0.000
Piped Water (*)	−0.630	0.000
Solid Waste Disposal (*)	−0.586	0.000

Note: (*) Positive indicators. Higher values are associated with lower risk of leptospirosis.

The 32 municipalities from the selected departments were divided into three groups based on the transformed leptospirosis rates: 12 municipalities had low rates (37.5%) (log-transformed rates range from 0 to 0.48); 10 municipalities had medium rates (31.3%) (log-transformed rates range from 0.55 to 0.97) and 10 municipalities had high rates (31.3%) (log-transformed rates range from 1.11 to 2.51). The total number of cases in the low rates group is 240 (0, 0, 0, 1, 3, 3, 5, 8, 18, 46, 77, 79); in the medium group is 169 (7, 7, 8, 8, 8, 16, 17, 20, 25, 53); and in the group of high rates is 592 (8, 14, 14, 39, 39, 43, 46, 100, 113, 176). Log-transformed rates in the low rates group range from 0 to 0.48 (0, 0, 0, 0.02, 0.09, 0.11, 0.12, 0.22, 0.23, 0.34, 0.42, 0.48); in the medium rates group range from 0.55 to 0.97 (0.55, 0.58, 0.6, 0.67, 0.78, 0.81, 0.86, 0.88, 0.97, 0.97) and in the high rates group range from 1.11 to 2.51 (1.11, 1.13, 1.14, 1.18, 1.21, 1.75, 1.91, 2.05, 2.28, 2.51). Throughout this aggregated analysis municipalities are the unit of analysis, and incidence rates and socioeconomic variables are the descriptors.

Table 3 shows the descriptive statistics (mean and standard deviation) of the percentages of the socioeconomic variables in the three groups of municipalities according to the leptospirosis log-transformed rates. There is a clear covariation between the socioeconomic indicators and the grouped rates. The municipalities with higher historical rates have poorer socioeconomic indicators. 

Table 4 contains the results of the two-stage cluster analysis performed in all municipalities of Chinandega, Leon, and Managua. Input variables for this analysis included the socioeconomic variables of the municipalities (unsatisfied basic needs, extreme poverty rate, illiteracy rate in population over 6 years, piped water and basic sanitation in the household). Leptospirosis rates were used as the criterion for assessing concurrent validity of the clusters of municipalities. 

**Table 3:** Mean and standard deviation of the percentages of the socioeconomic variables in the grouped municipalities by log-transformed rates of leptospirosis between 2004 and 2010.

Number of Observations	Group Rates		UBN Quality Household	UBN Sanitary Services	UBN Crowding	UBN Education	Extreme Poverty	Illiteracy	Piped Water	Solid Waste Disposal
*N* = 12	Low Rates (0–0.48)	Mean	36.8	15.9	22.2	6.7	24.6	10.2	50.3	34.3
		SD	11.4	8.3	5.0	1.6	5.1	1.3	18.6	22.7
*N* = 10	Medium Rates (0.55–0.97)	Mean	58.7	33.3	30.1	9.9	42.1	17.6	22.1	7.4
		SD	12.2	11.5	13.8	.8	5.5	1.4	15.4	6.5
*N* = 10	High Rates (1.11–2.51)	Mean	64.8	37.2	37.7	11.4	42.9	20.3	15.4	3.0
		SD	11.1	9.9	8.5	2.9	9.3	1.6	8.3	1.4

Note: SD: Standard Deviation.

**Table 4:** Characterization and validation of clusters of municipalities (mean values).

Variables	Clusters of Municipalities		
	1	2	3
UBN Quality Household	70.5%	52.1%	34.6%
UBN Sanitary Services	42.6%	27.1%	14.1%
UBN Crowding	42.1%	24.3%	21.6%
UBN Education	11.7%	9.3%	6.6%
Extreme Poverty	47.4%	35.9%	24.1%
Illiteracy	20.0%	17.0%	11.0%
Piped Water	10.6%	27.4%	53.5%
Solid Waste Disposal	2.5%	8.0%	36.9%
Rates of Leptospirosis per 10 thousand	3.1	1.4	0.2

Cluster of municipalities 1 has the highest leptospirosis rate and the worst socioeconomic conditions as given by all indicators, in contrast with cluster of municipalities 3 which presents the lowest rate and the best socioeconomic conditions. Cluster of municipalities 2 has intermediate values for all indicators. The variables are consistently distributed among the clusters of municipalities. Transformed rates of leptospirosis are in perfect agreement with the classification given by the rest of the variables.

**Figure 1:**
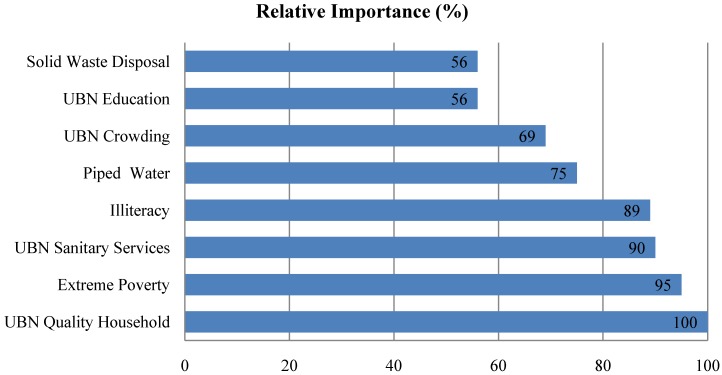
Relative importance of the variables in the definition of the clusters of municipalities.

The variables were ordered according to their relative importance in defining the clusters of municipalities, as shown in Figure 1, in which the most relevant variable (unsatisfied basic needs for quality of the household) was assigned an importance of 100% and the remaining variables were expressed as a percentage of that reference. Higher extreme poverty rates (95%), greater unsatisfied basic needs for sanitary services (90%) and higher illiteracy rates (89%) are also important in the definition of the clusters. 

Groups were formed after fitting a classification tree and finding optimal cut-off points. Since the objective of this analysis was to create an index of vulnerability and not to measure the impact of socioeconomic indicators on leptospirosis, it was not necessary to consider the assumption of uncorrelated socioeconomic indicators. If two correlated socioeconomic indicators are both present in a given municipality their joint effect must be considered to assess the degree of vulnerability of that municipality. The LVI is a latent variable which takes into account the polarity of the variables in the allocation of the signs and was obtained as a combination of the variables in which the relative importance or coefficient is used as the weighed factor.

LVI characterizes municipalities which in turn are stratified by departments. .When this LVI was applied to the departments of Chinandega, Leon, and Managua, it led to the classification of the all 32 municipalities of these departments, which explains a high percentage of the variability of the municipal rates of leptospirosis. This relationship is shown in Table 5.

**Table 5:** Mean and standard deviations of the local vulnerability index according to groups given by the log-transformed rates.

Groups	Mean	N	Standard Deviation
Low rates	36.7	12	46.5
Medium rates	134.4	10	48.9
High rates	158.4	10	35.6
Total	105.3	32	69.5

Notes: R = 0.75; R^2^ = 0.56; % of explained variance = 56%.

The LVI differs greatly between groups of municipalities classified according to their rates. The difference is particularly large among the municipalities with low rates *versus* those with intermediate and high rates of leptospirosis. A linear model explaining the classification according to the log-transformed rates from LVI explains 56% of the variability. For a single classification factor, this percentage can be considered to be high. It means that the proportion of variability in log-transformed rates is greater between groups that within groups.

The numbers of municipalities at the low, medium and high rate groups are 12, 10 and 10, respectively. Simple descriptive statistics was used to show the gaps in the vulnerability index, particularly between the first group (low rate) and the other two groups. However, the R^2^ was reported to show a strikingly high proportion on inter-group variability as compared to total variability. No inferential statistical analyses were performed. 

**Figure 2:**
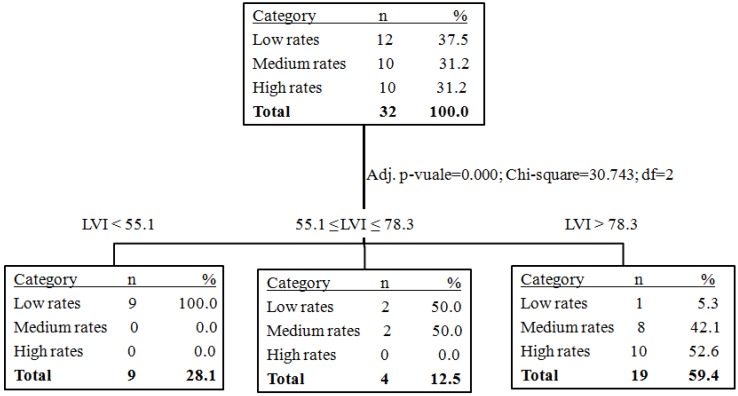
Classification tree according to the local vulnerability index (LVI).

**Figure 3:**
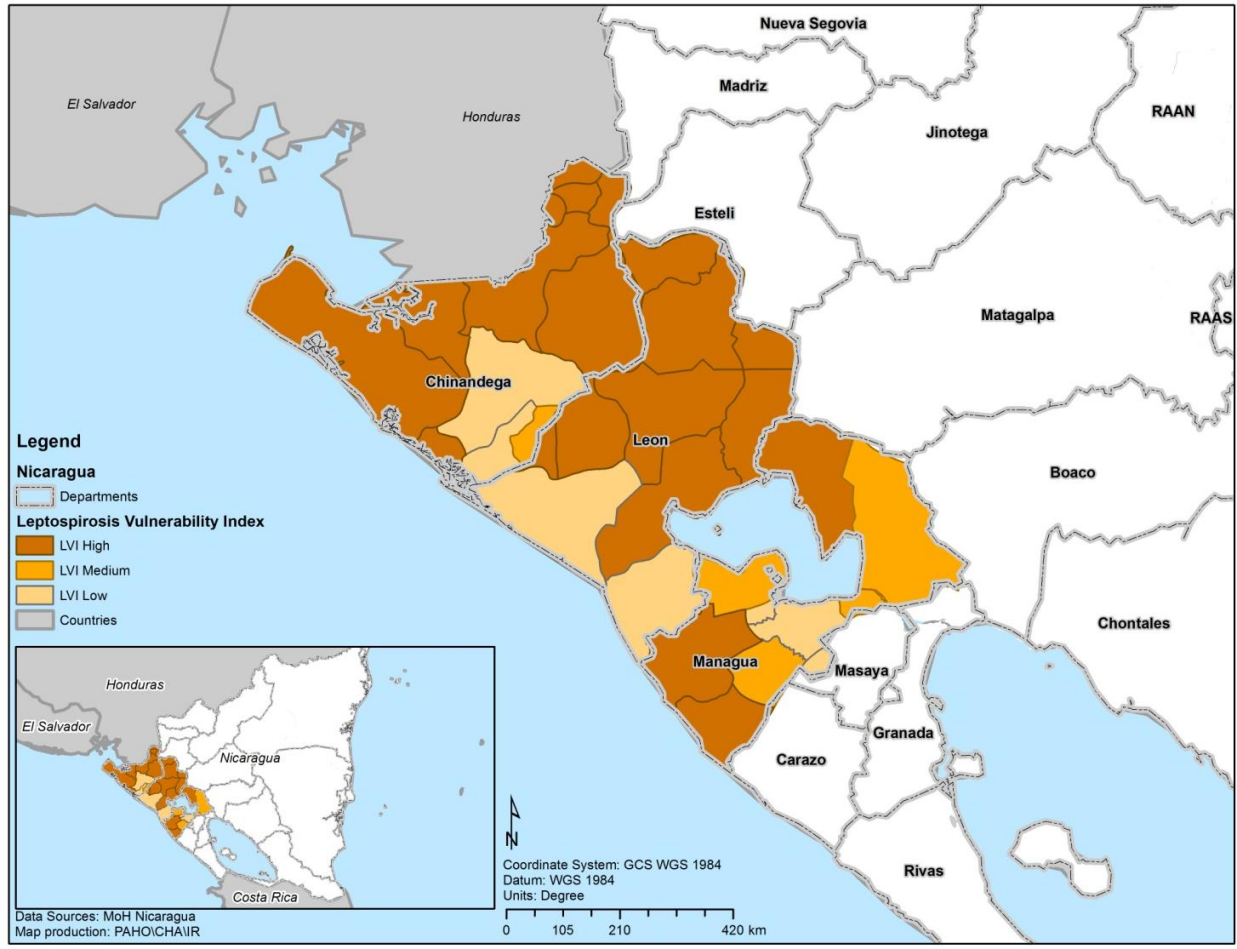
Municipalities of Chinandega, Leon, and Managua according to the local vulnerability index (LVI).

The classification tree (Figure 2) identifies optimal cutoffs for LVI which are also the range of the values for the index for the three categories. In municipalities with LVI ≤ 55.1, all municipalities had low leptospirosis rates; in municipalities with 55.1 ≤ LVI ≤ 78.3, 50% of them had low rates and 50% intermediate rates; and in municipalities with LVI > 78.3, 94.7% of them had high (52.6%) or intermediate rates (42.1%). The municipalities with high LVI (>78.3, 94.7%) are the most vulnerable for leptospirosis according to this index. The classification tree provides objective criteria for the concurrent validity of LVI as an indicator of vulnerability for risk areas and outbreak situations. According to the classification tree, among the 32 municipalities studied, 19 were classified as high LVI (Figure 3). Those municipalities should be given priority for intervention, prevention and control of leptospirosis.

The results of this study provide evidence which supports the hypothesis that municipalities with lower socioeconomic status (higher poverty rates, greater unsatisfied basic needs and worse education conditions) have higher leptospirosis rates. The LVI can be used to sort the municipalities along a latent axis that correlates highly with disease rates and can thus be used to target interventions or preventive actions in areas of risk for leptospirosis. 

Local interventions for leptospirosis prevention and control were described in the Intersectoral National Leptospirosis Plan for Nicaragua, developed by national authorities in 2011, and several interventions have already being implemented in the country [31]. In order to respond to possible outbreaks, the country has coordinated communities and trained health professionals in laboratory and clinical case management of leptospirosis, as well as promoted early warning of febrile cases [32]. Departments are also taking action in the prevention of the disease: the Department of Leon developed a departmental emergency council against leptospirosis and strengthened epidemiological surveillance and diagnosis [33]. In the Department of Chinandega leptospirosis prevention is carried out through health promotion activities and education about protection for risk groups and animals [34]. In addition, animal antibiotic treatment schemes have been applied to selected livestock and rodent trapping has been used to characterize house infestations and sources of infection in certain areas to determine carriers and shedders of the bacteria [35].

This study underpins the conceptual duality of the causes of cases and the causes of outbreaks. The circumstances (altogether explained by physical, environmental and ecological factors) that determine outbreaks do not have the same effect in all territorial units studied. There are disease aggregation factors, since the disease is not distributed randomly in the areas most exposed to environmental agents.

**Figure 4:**
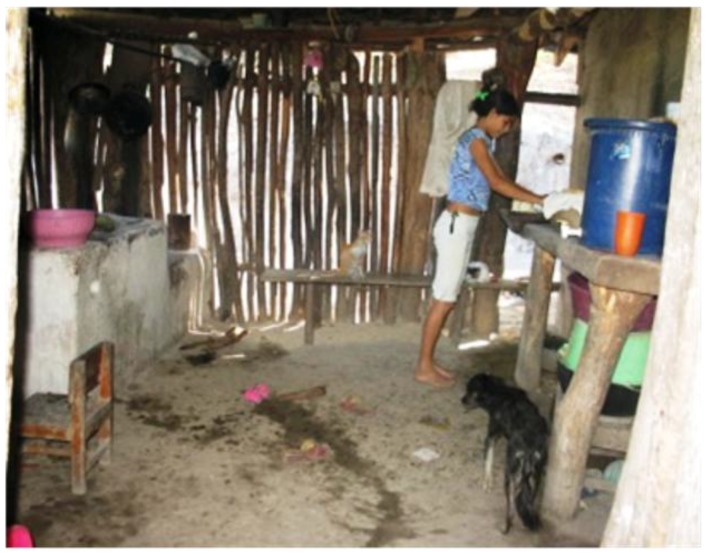
Typical household of low-income rural area in Nicaragua.

Note: Photo: Gilberto Moreno.

This analysis shows the importance of socioeconomic factors for the vulnerability to leptospirosis outbreaks. The unsatisfied basic need for quality of the household, as well as the UBN for access to sanitary services and the extreme poverty rates are the basic components of the vulnerability index. These findings are consistent with the verbal reports during field work in Nicaragua, in which local experts reported that outbreaks coincided with harvest periods and heavy rains, and that farmers from low-income areas have the habit of putting animals and crops inside the household to protect them from the rain. The months of June to October are the time period with the highest precipitation in the Pacific Coast of Nicaragua, with the peak in September. The number of leptospirosis cases significantly increases during this time, compared to the dry season in the beginning of the year it is about ten times higher [4]. In dwellings without adequate floors, e.g., permeable instead of concrete floors, animal urine in a muddy floor with rain turns into a wet environment, which increases the risk of leptospirosis transmission (Figure 4). Similarly, crops stored improperly inside dwellings built with vulnerable materials can attract infected wild rodents and also increase the risk of transmission. In a previous study conducted in Nicaragua, a significant association between leptospirosis rates and percentage of rural population was found, which supports this finding [4]. 

**Figure 5:**
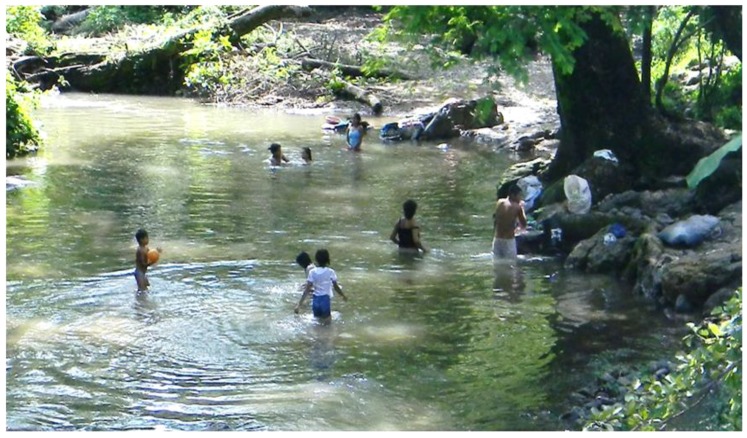
The use of rivers or creeks for different purposes in low-income rural areas in Nicaragua.

Note: Photo: Gilberto Moreno.

**Figure 6:**
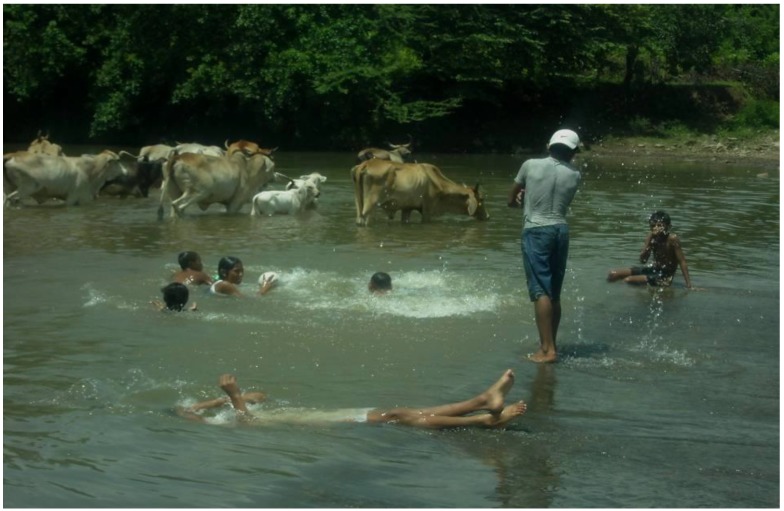
Humans and animals using the river in rural areas in Nicaragua.

Note: Photo: Octavio Chávez

As for the UBN related to sanitary services, the common practice of using creeks or rivers to bath and to wash clothes may increase the risk of becoming infected by the bacteria in the water (Figure 5). Local observations also suggest that in rural areas of Nicaragua animals are taken to rivers to drink water and in case they are infected they could contaminate the water through urine (Figure 6). The individual risk factor of bathing in creeks was found to be statistically significant in the Nicaragua outbreak investigation of 1995 [23]. A previous study confirms that rural households that lack access to piped water and basic sanitation services may use the river as a source of water and to wash their clothes, at the same time they may take their animals to bath and drink water; these activities are significant risk factors for leptospirosis [36]. 

Worldwide the infection with *Leptospira* occurs in approximately 160 mammalian species, each serovar (basic systematic unit) has its preferred animal host [8]. The presence of 20 serovars in Nicaragua was characterized by serology in several species such as bovines, swine, equines and canine [32]. Animals, such as cattle, that shed the bacteria for longer time periods and release large amounts of urine are very common in rural regions of Nicaragua. This may be one of the major problems in the transmission cycle of leptospirosis in these areas, which need to be further investigated in the future. In addition, leptospirosis infection in livestock may also have an economic impact for the country, since it can affect milk production and cause abortions [8]. Additional studies are also needed to better understand the transmission cycles of leptospirosis in different settings of Centro America such as rural communities, villages and slums, like the currently ongoing study in Chile [37].

Rodents are well known animals related to the leptospirosis transmission cycles in rural and urban environments [8,38,39]. Nicaragua’s National Intersectoral Leptospirosis Plan includes rodent control activities, which are being developed and implemented in risk areas [32]. However, the only well document study that includes animal trapping and isolation of the bacteria was conducted in 1995 during Nicaragua’s first outbreak investigation, where high percentages of the bacteria was found in tested rodents and isolated from urine of domestic animals [23]. Further studies about leptospirosis transmission among different animal species and the impact of strategies such as rodent control and cattle vaccination can be useful for the National Plan.

Previous studies have demonstrated that household environment is an important transmission determinant in the urban slum settings, in addition close contact with garbage and sewage are significant risk factors in leptospirosis transmission [3,10,11]. The presence of improperly storage food, garbage and sewage encourage the proliferation of rodents, also deficiencies in the sanitation infrastructure are environmental sources of *Leptospira* transmission and can therefore increase the risk of leptospirosis [3,10]. 

The methodological approach to create a vulnerability index, in spite of already knowing which municipalities have higher incidence rate, was chosen to support future decision making and maybe using the index for other similar scenarios. Models are created on theoretical grounds and by using observed data, but they are not 100% accurate. In fact, researchers choose between models on the basis of their observed accuracy. In practice, no classification tree or any other classifier can be expected to predict with 0 percent misclassification. 

It should be noted that these results were obtained from an aggregated analysis using the clusters of the municipalities as the unit of analysis, and additional local studies are necessary using households and individuals as the units of analysis. Commonly associated with this type of study is the ecological fallacy [40]. However, the purpose of this study is to identify the most vulnerable areas to support interventions in the countries that are usually divided by administrative areas, using existing epidemiological and census data from the country’s information system. 

## 4. Conclusions 

The contributions of this study are manifested in three aspects. In a practical sense, these results support a more objective focus on the preventive and response actions to outbreaks of leptospirosis. As the municipalities with high LVI (>78.3, 94.7%) were nominal identified in the application of this index in the field in Nicaragua, they are the most vulnerable municipalities for leptospirosis, according to this study. Decision makers can use these results as evidence to appoint priority areas for interventions and allocate resources such as medications, hospital beds, equipment and health personnel to respond to outbreaks. The municipalities with highest LVI were identified, which should have highest priority for intervention, followed by municipalities with medium LVI rates, since a previous study has shown that there is environmental risk in these departments. This LVI could be examined jointly with previous epidemiological methodology for measuring critical areas already applied to Nicaragua, taking into consideration the country’s interventions outlined in the Intersectoral National Leptospirosis Plan and those that are already being implemented in Nicaragua, in order to provide a combination of tools for evidence based decision making [4,31].

Conceptually, the underlying distinction between risk (given mainly by environmental factors) and vulnerability to risk (given mainly by socioeconomic conditions) was shown as fundamental and this distinction also applies to the “causes of outbreaks” and “causes of cases”. Methodologically, a vulnerability index was developed and validated with a high capacity for identifying vulnerable municipalities in areas of risk. Finally, we have confirmed the importance of unsatisfied basic needs in relation to the construction material conditions of the household, unsatisfied basic needs for access to sanitary services, extreme poverty and illiteracy rates as a basis for targeting prevention and control actions against leptospirosis.

Analyzing socioeconomic variables available in secondary sources could be used as a “proxy” to demonstrate the relationship between social determinants of health and leptospirosis cases. Studying the inequality distribution of the social determinants of health in addressing infectious diseases shows the importance of applying an intersectoral approach in handling the problem, promoting access to health care to the cases, implementing prevention intersectoral strategies in high risk areas and also improving living conditions. 

This study also serves as a foundation for possible extrapolation of this methodological approach in identifying risk areas by their drivers to other countries in the region, as well as to other diseases. If surveillance systems for certain diseases are not yet well established in a country and the drivers for those diseases are known, this methodology could serve as evidence-base to strengthen disease surveillance in those areas. The application of the methodology in other scenarios would need to take into account contextual factors, and on the other hand, peculiar traits of disease and its distribution. However, the crucial distinction between causes of outbreaks and determinants of cases is likely to be valid although it would need further validation by using households and individuals as the unit of analysis.

## References

[1] World Health Organization (2010). Report of the First Meeting of the Leptospirosis Burden Epidemiology Reference Group.

[2] World Health Organization (2003). Human Leptospirosis: Guidance for Diagnosis, Surveillance and Control.

[3] Lau C.L., Smythe L.D., Craig S.B., Weinstein P. (2010). Climate change, flooding, urbanisation and Leptospirosis: Fuelling the fire?. Trans. R. Soc. Trop. Med. Hyg..

[4] Schneider M.C., Nájera P., Aldighieri S., Bacallao J., Soto A., Marquiño W., Altamirano L., Saenz C., Marin J., Jimenez E. (2012). Leptospirosis outbreaks in nicaragua: identifying critical areas and exploring drivers for evidence-based planning. Int. J. Environ. Res. Public Health.

[5] McMichael A.J. (2001). Human Frontiers, Environments and Disease: Past Patterns, Uncertain Futures.

[6] Aron J.L., Patz J.A. (2001). Ecosystem Change and Public Health: A Global Perspective.

[7] Kingscote B.F. (1970). Correlation of bedrock type with the geography of Leptospirosis. Can. J. Comp. Med..

[8] Acha P., Szyfres B. (2003). Zoonoses and Communicable Diseases Common to Man and Animals: Bacterioses and Mycoses.

[9] Lau C.L., Dobson A.J., Smythe L.D., Fearnley E.J., Skelly C., Clements A.C., Craig S.B., Fuimaono S.D., Weinstein P. (2012). Leptospirosis in American Samoa 2010: Epidemiology, environmental drivers, and the management of emergence. Amer. J. Trop. Med. Hyg..

[10] Reis R.B., Ribeiro G.S., Felzemburgh R.D., Santana F.S., Mohr S., Melendez A.X., Queiroz A., Santos A.C., Ravines R.R., Tassinari W.S. (2008). Impact of environment and social gradient on Leptospira infection in urban slums. PLoS Negl. Trop. Dis..

[11] Barcellos C., Sabroza P.C. (2001). The place behind the case: Leptospirosis risks and associated environmental conditions in a flood-related outbreak in Rio de Janeiro. Cadernos de Saúde Pública.

[12] Bhardwaj P., Kosambiya J., Desai V. (2008). A case control study to explore the risk factors for acquisition of Leptospirosis in Surat city, after flood. Indian J. Med. Sci..

[13] Bovet P., Yersin C., Merien F., Davis C.E., Perolat P. (1999). Factors associated with clinical Leptospirosis: A population-based case-control study in the Seychelles (Indian Ocean). Int. J. Epidemiol..

[14] Dias J.P., Teixeira M.G., Costa M.C.N., Mendes C.M.C., Guimarães P., Reis M.G., Ko A., Barreto M.L. (2007). Factors associated with Leptospira sp. infection in a large urban center in northeastern Brazil. Rev. Soc. Brasil. Med. Trop..

[15] World Health Organization Closing the Gap: Policy into Practice on Social Determinants of Health. Proceedings of the World Conference on Social Determinants of Health.

[16] World Health Organization and Commission in Social Determinants of Health (2008). Closing the Gap in a Generation: Health Equity through Action on the Social Determinants of Health.

[17] World Health Organization Outcome of the World Conference on Social Determinants of Health. Resolution WHA65.8. Proceedings of the Sixty-Fifth World Health Assembly.

[18] World Health Organization The Helsinki Statement on Health in All Policies. Proceedings of the 8th Global Conference on Health Promotion.

[19] Smith L.C., Haddad L.J. (2000). Explaining Child Malnutrition in Developing Countries: A Cross-country Analysis.

[20] Instituto Nacional de Información de Desarrollo (INIDE) (2005). VIII Censo de Población y IV de Vivienda. http://www.inide.gob.ni/censos2005/CifrasMun/tablas_cifras.htm.

[21] Pan American Health Organization (1998). Impacto del Huracán Mitch en Centro América. Boletin Epidemiol..

[22] Centers of Disease Control and Prevention (1995). Outbreak of acute febrile illness and pulmonary hemorrhage—Nicaragua, 1995. Morbid. Mortal. Wkly. Rep..

[23] Trevejo R.T., Rigau-Pérez J.G., Ashford D.A., McClure E.M., Jarquín-González C., Amador J.J., José O., Gonzalez A., Zaki S.R., Shieh W.-J. (1998). Epidemic Leptospirosis associated with pulmonary hemorrhage—Nicaragua, 1995. J. Infect. Dis..

[24] Ministry of Health of Nicaragua Sistema Nicaragüense de Vigilancia Epidemiológica Nacional (SISNIVEN). http://www.minsa.gob.ni/index.php?option=com_remository&Itemid=52&func=select&id=1686.

[25] Comisión Económica para América Latina y el Caribe (CEPAL) Indicadores de Pobreza: Necesidades Básicas Insatisfechas. http://www.eclac.cl/cgi-bin/getprod.asp?xml=/esalc/noticias/paginas/4/12754/P12754.xml&xsl=/esalc/tpl/p18f.xsl&base=/esalc/tpl/top-bottom.xsl.

[26] General Division of Public Health Surveillance, Ministry of Health of Nicaragua General Information. http://www.minsa.gob.ni/.

[27] Feres J.C., Mancero X. (2001). El Método de las Necesidades Básicas Insatisfechas (NBI) y sus Aplicaciones en América Latina.

[28] Bacher J. (2000). A probabilistic clustering model for variables of mixed type. Qual. Quant..

[29] Watson C.G., Juba M.P., Manifold V., Kucala T., Anderson P.E. (1991). The PTSD interview: Rationale, description, reliability, and concurrent validity of a DSM-III based technique. J. Clin. Psychol..

[30] Sudat S.E., Carlton E.J., Seto E.Y., Spear R.C., Hubbard A.E. (2010). Using variable importance measures from causal inference to rank risk factors of schistosomiasis infection in a rural setting in China. Epidemiol. Perspect. Innov..

[31] (2011). Plan de Trabajo Interinstitucional de Abordaje Integral a la Leptospirosis.

[32] Sánchez E. Epidemiological Situation of Leptospirosis in Nicaragua and the Intersectoral Plan. http://www.paho.org/hq/index.php?option=com_docman&task=doc_view&gid=19933&Itemid=.

[33] Moreno G. Experience in Controlling Outbreaks of Leptospirosis in Leon. http://www.paho.org/hq/index.php?option=com_docman&task=doc_view&gid=19927&Itemid=.

[34] Chávez O. Experience in Controlling Outbreaks of Leptospirosis in Chinandega. http://www.paho.org/hq/index.php?option=com_docman&task=doc_view&gid=19926&Itemid=.

[35] Soto A. Preliminary Analysis of the Inter-Institutional Plan of Comprehensive Approach to Leptospirosis. http://www.paho.org/hq/index.php?option=com_docman&task=doc_view&gid=19924&Itemid=.

[36] Ashford D.A., Kaiser R.M., Spiegel R.A., Perkins B.A., Weyant R.S., Bragg S.L., Plikaytis B., Jarquin C., Reyes J.D.L., Amador J.J. (2000). Asymptomatic infection and risk factors for Leptospirosis in Nicaragua. Amer. J. Trop. Med. Hyg..

[37] Muñoz-Zanzi C., Mason M., Encina C., Gonzalez M., Berg S. (2014). Household characteristics associated with rodent presence and Leptospira infection in rural and urban communities from southern Chile. Amer. J. Trop. Med. Hyg..

[38] Hartskeerl P., Terpstra W. (1996). Leptospirosis in wild animals. Vet. Quart..

[39] Faria M.T., Calderwood M.S., Athanazio D.A., McBride A.J., Hartskeerl R.A., Pereira M.M., Ko A.I., Reis M.G. (2008). Carriage of *Leptospira interrogans* among domestic rats from an urban setting highly endemic for leptospirosis in Brazil. Acta Tropica.

[40] Porta M. (2008). A Dictionary of Epidemiology.

